# Cervical Stiffness Evaluated *In Vivo* by Endoflip in Pregnant Women

**DOI:** 10.1371/journal.pone.0091121

**Published:** 2014-03-06

**Authors:** Lene Hee, Donghua Liao, Puk Sandager, Hans Gregersen, Niels Uldbjerg

**Affiliations:** 1 Department of Obstetrics and Gynecology, Aarhus University Hospital, Aarhus, Denmark; 2 Institute of Clinical Medicine, Aarhus University, Aarhus, Denmark; 3 Mech-Sense, Department of Gastroenterology and Surgery, Aalborg University Hospital, Aalborg, Denmark; 4 Giome Center, College of Bioengineering, Chongqing University, Chongqing, China; 5 Giome Institute, GIOME FZE, Ras Al Khaimah, United Arab Emirates; National Cancer Institute, United States of America

## Abstract

**Objective:**

To determine the stiffness of the pregnant uterine cervix in vivo.

**Method:**

Five women in early pregnancy and six women in late pregnancy were included. The EndoFlip is a 1-m-long probe with a 12-cm-long bag mounted on the tip. The tip of the probe was inserted into the cervical canal. Sensors spaced at 0.5-cm intervals along the probe were used to determine 16 serial cross-sectional areas of the bag. The diameter of the cervical canal could thereby be determined during inflation with up to 50 ml saline solution. Tissue stiffness was calculated from the geometric profiles and the pressure-strain elastic modulus (EP) at each sensor site. Three parts of the cervix were defined: the uterus-near part, the middle and the vaginal part. The EP_max_ was defined as the highest EP detected along the cervical canal.

**Results:**

The EP_max_ was always found in the middle part of the cervix. The median EP_max_ was 243 kPa (IQR, 67–422 kPa) for the early pregnant women and 5 kPa (IQR, 4–15 kPa) for those at term. In the early pregnant women the stiffness differed along the cervical length (p<0.05) whereas difference along the cervix was not found for late pregnant women. A positive correlation coefficient (Spearman’s rho) was established between the EPs of the uterus-near and the middle part (0.84), between the vaginal and the middle part (0.81), and between the uterus-near and the vaginal part (0.85).

**Conclusion:**

This new method can estimate the stiffness along the cervical canal in vivo. This method may be useful in the clinical examination of the biomechanical properties of the uterine cervix.

## Introduction

Most methods used for labor induction and estimation of the risk of preterm delivery exclusively evaluate the vaginal part of the uterine cervix, such as digital evaluations using the Bishop’s score [1], [2]. This also applies to more objective methods, including collascope measuring cross-linking of collagen from light-induced fluorescence [3], [4] and devices measuring tissue deformation during aspiration [5] or compression [6]. These methods rest on the assumption that the stiffness (or distensibility) of the uterine-near part and the vaginal part of the uterine cervix are similar.

However, studies have documented that axial variation actually exists along the cervix. Histological studies found more than 29% smooth muscle in the uterine-near part compared with only 6% in the vaginal part [7], [8]. Furthermore, ultrasound scanning during pregnancy has shown that cervical shortening is often associated with cervical funneling [9]. Both observations suggest an increased distensibility or lower stiffness of the uterine-near part of the cervix. Elastography examinations suggest an increased compressibility of the vaginal part of the cervix, a finding that may not be valid as the vaginal part of the uterine cervix was more compressed by the transducer than the uterine-near part [10]. Thus data obtained with conventional methods seem to be biased because they examine only the vaginal part of the uterine cervix. Other parts of the uterine cervix may also have an important bearing on diseases and diagnostics.

The aim of the present study is to compare tissue stiffness within the entire uterine cervix during early and late pregnancy with the use of the Endolumenal Functional Imaging Probe (EndoFlip, Crospon Inc., Galway, Ireland), which determines cervical cross-sectional areas (diameters) at 16 sensors along a probe inserted into the cervical canal. If axial variation in the diameters or stiffness parameters derived from the diameter-pressure relations along the cervical canal can be demonstrated, then the validity of the other above mentioned methods needs reevaluation.

## Materials and Methods

A pilot study of nine pregnant women was conducted prior to the study described below. Different distensions of the bag (10–50 ml) were evaluated. A distension volume of 50 ml was selected as the cervix of the term-pregnant women would otherwise not be dilated mechanically by a smaller volume. Furthermore, the maximum pressure of 150 mmHg inside the bag, fixed from the manufacturer for safety reasons, was achieved with this volume.

Fifteen pregnant women were included in this study: Seven in early pregnancy and eight women in late pregnancy. Inclusion criteria were pregnant women admitted for surgical abortion in the first trimester, and women admitted for elective caesarian section at term, where an uncomplicated procedure was expected. Exclusion criteria were prior conization and prior preterm delivery. Four experiments (two in early pregnancy and two in late pregnancy) were omitted because the probe could not be placed correctly in the cervix, mainly because of difficulties with passing the orificium internum with the probe. This was noticed from the spatial-temporal diameter plot and by too many sensors visible outside the orificium externum. To overcome this problem the cervical canal was passed with a uterine sound prior to the placement of the probe in the following experiments. The final study group consisted of five women in early pregnancy (gestational age 7^+1^–8^+5^ weeks) and six women in late pregnancy (gestational age 38^+4^–40^+3^ weeks). The early-pregnant women were examined just before surgical abortion and the term-pregnant women within 15–20 minutes after the elective cesarean section.

The study was approved by the Scientific Ethics Committee of the Aarhus County (no. 20100149) and the Danish Data Surveillance Authority (2007-58-0010). Written and verbal informed consent was obtained from all participants.

**Figure 1:**
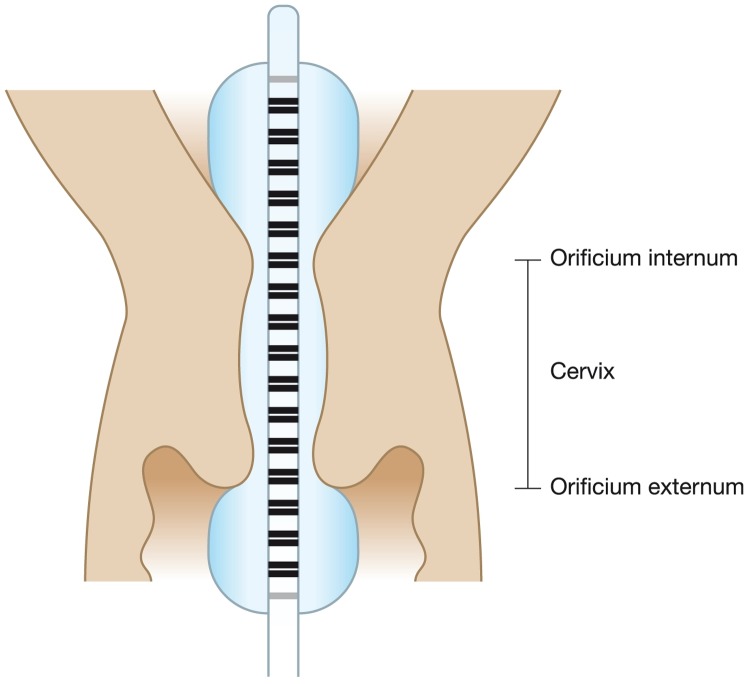
Illustration of the probe in the uterine cervical canal. The tip of the probe is placed in the uterine cavity, with the middle two thirds of the probe in the cervical canal leaving 2–3 sensors visible outside the canal protruding into the vagina. The two gray lines represent the excitation electrodes whereas the 16 black sensors detect 16 cross-sectional areas along the probe.

**Figure 2:**
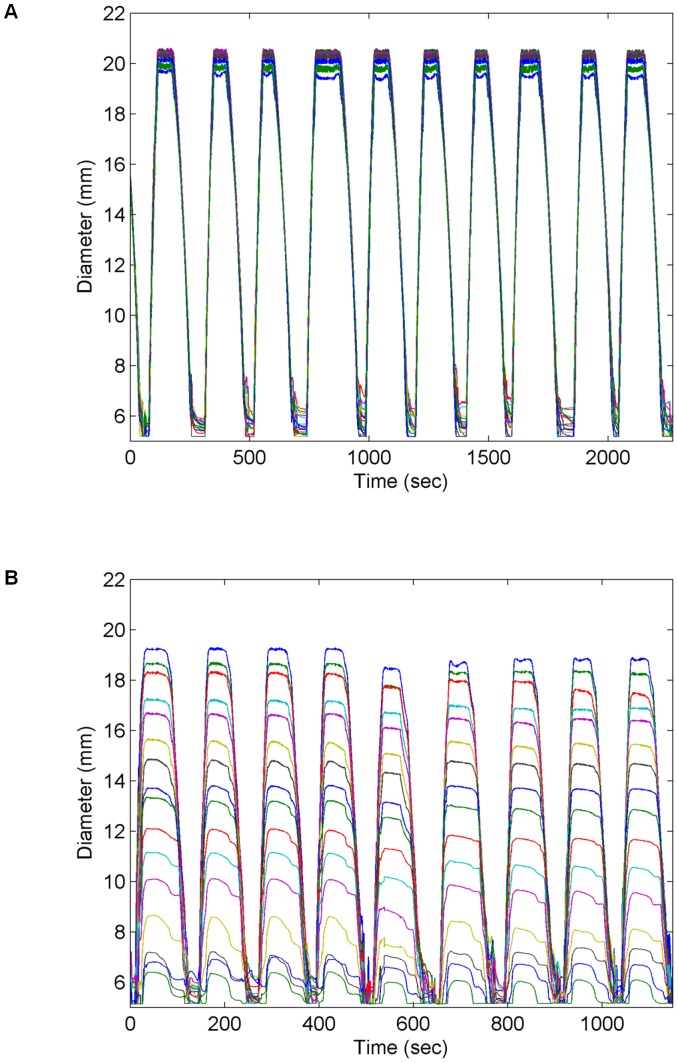
Validation data from testing in two phantoms. Each colored line represents the diameter data obtained from each sensor. The bag was inflated and deflated several times in a cylinder-shaped phantom with the diameter 20.8 mm (A) and in a funnel-shaped phantom with diameters ranging from 5–19 mm (B). The tracings demonstrate that the diameter measurements are accurate and reproducible.

The EndoFlip is 1-m-long probe (diameter 2.8 mm) with a 12-cm-long cylindrical polyester urethane bag mounted on the tip. The probe is connected to an infusion pump that fills saline solution into and out of the bag. The probe measures the cross-sectional areas of the bag (corresponding to diameters of 5–25 mm) at 16 serial locations 5 mm apart, and a pressure transducer determines the pressure inside the bag (Figure 1). The cross-sectional area at each of the 16 locations was estimated by the field gradient principle, which is based on electrical impedance measurements [11]. Each of the 16 sensors consists of two detection electrodes which measure the voltage difference induced by a current (I) generated by two excitation electrodes, placed on the probe at each end of the bag (Figure 1). The voltage difference (V) between the detection electrodes can be expressed as V = R x I, where R, the electrical impedance of the saline solution, can be expressed as d x σ^−1^ x CSA ^−1^; d is the distance between the detecting pair of electrodes, σ is the conductivity of the saline solution and CSA is the cross-sectional area. As d, σ and I are constants, V is inversely proportional to the CSA of the bag; thereby, the CSA can be determined by proper calibration [12]–[14]. The CSA is converted to diameter assuming the lumen is cylindrical. Analyses and data display were done with Matlab R14, Mathworks Inc. In vitro experiments showed a mean deviation from the true values of 3.1% (SD 0.5%) in tube shaped phantoms and 4.1% (SD 1.9%) in a funnel shaped phantom (Figure 2).

A transvaginal ultrasound scan of the uterine cervix was performed and the participants had the cervix visualized by the use of a sterile vaginal speculum. A double tooth tenaculum was attached to the anterior lip of the cervix and the cervical canal was passed by a uterine sound before the probe was inserted. The tip of the probe was inserted into the cervical canal until only 2–5 sensors were visible outside the orificium externum, ensuring that the tip of the probe was placed in the uterine cavity (Figure 1). Then infusion was started at a volume rate of 12 ml/min up to a maximum volume of 50 ml which was kept constant for two minutes before deflation. The recorded pressure, the volume in the bag, and the diameters along the 16 sensors were used for all the data analysis. To obtain a smoothed diameter distribution along the cervical canal, a one-dimensional interpolation along 16 sensors with calculated diameter in every 0.5 mm was applied.

The stiffness of the uterine cervix was expressed as the pressure-strain elastic modulus (EP) [15]. The EP was calculated as:
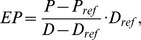
where P, P_ref_, D and D_ref_ is pressure during inflation, pressure at the reference volume, the corresponding diameter during inflation and the diameter at the reference volume, respectively. The volume of 5 ml was selected for D_ref_ where the diameter of the bag started to increase. For each recording we defined three parts representing the uterus-near part, the middle part and the vaginal part of the cervix. The three parts were identified by knowing the cervical length from the ultrasound scans and the plots described above. The middle part of the cervix was defined as the locations with the highest EP (EP_max_). As the highest EP was never found at the ends of the cervical canal, a uterine-near (two to three sensors) and a vaginal part (two to three sensors) of the cervix were defined on each side of middle part. Each part includes two to three locations with 2.5 mm in between, and data for every part were averaged from calculations in these two to three locations. In three early-pregnant women the cervical length was not be obtained. In these cases we estimated the middle part of the uterine cervix from the spatial-temporal diameter plots. The middle locations of the uterus-near third and vaginal third of the canal were chosen to ensure the measurements were in the cervical canal and not in the uterus or the vagina.

### Statistical Analysis

The measurements in the tubes were expressed as means with a standard deviation. The mean EP of the two to three points for each part of the uterine cervix was selected for the calculations. The EPs were expressed as medians with interquartile ranges (IQR). The data were log-transformed in order to compare the three cervical parts for each group by one-way ANOVA and to compare the two groups by an unpaired t-test. The association between the different parts of the cervix was analyzed by Spearman’s rho.

For P-value <0.05 was considered statistically significant in two-tailed tests. STATA statistical software package, version IC10 (StataCorp., College Station, TX, USA) was used for the analyses.

## Results

**Figure 3:**
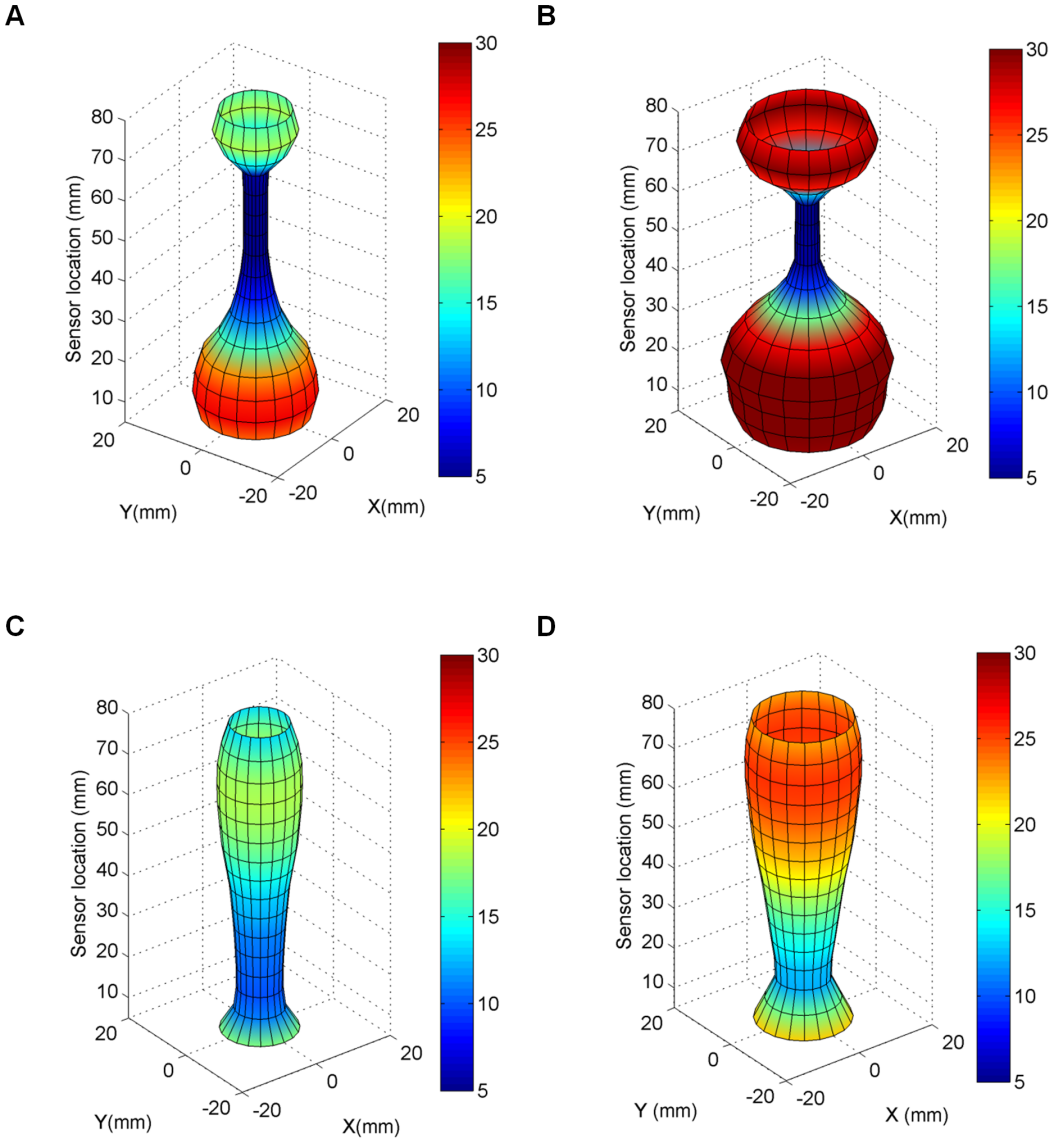
Semi-three dimensional plot. Shape of the uterine cervix for an early-pregnant woman during distension volume 25 ml (A) and 50 ml (B), and for a term-pregnant woman (C) 25 ml and (D) 50 ml. The geometry is clearly different between the two women.

**Figure 4:**
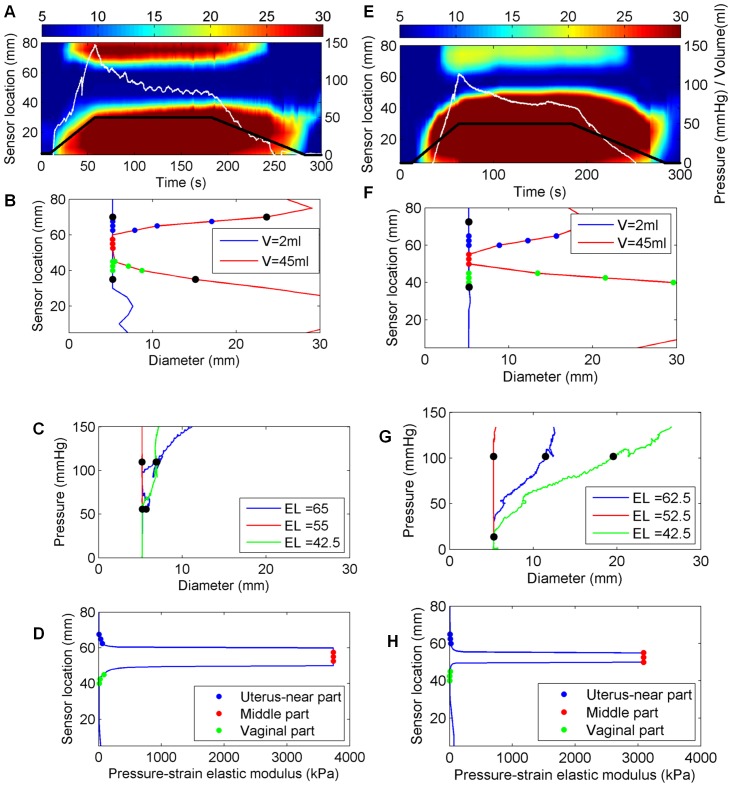
Geometric and biomechanical properties of the uterine cervix of an early-pregnant woman (left column, A–D) and term-pregnant woman with an unripe cervix (right column E–H). A and E) Spatio-temporal diameter plots. The black line illustrates the volume of the bag (ml), the white line the pressure inside the bag (mmHg), and the colors spanning from blue to red illustrate the magnitude of diameters obtained in the measurement area. B and F) The cervical canal configuration generated at distension volume 2 ml (blue line) and 45 ml (red line). The black dots represent the internal and external cervical os of the uterine cervix. C and G) Pressure-diameter plot. The pressure-diameter relationship for three locations representing the uterus-near part (blue line), the middle part (red line), and the vaginal part (green line) of the uterine cervix. The black dots represent the linear part of the curve. D and H) Pressure-strain elastic modulus plot. The pressure-strain elastic modulus distribution along the cervical canal.

**Figure 5:**
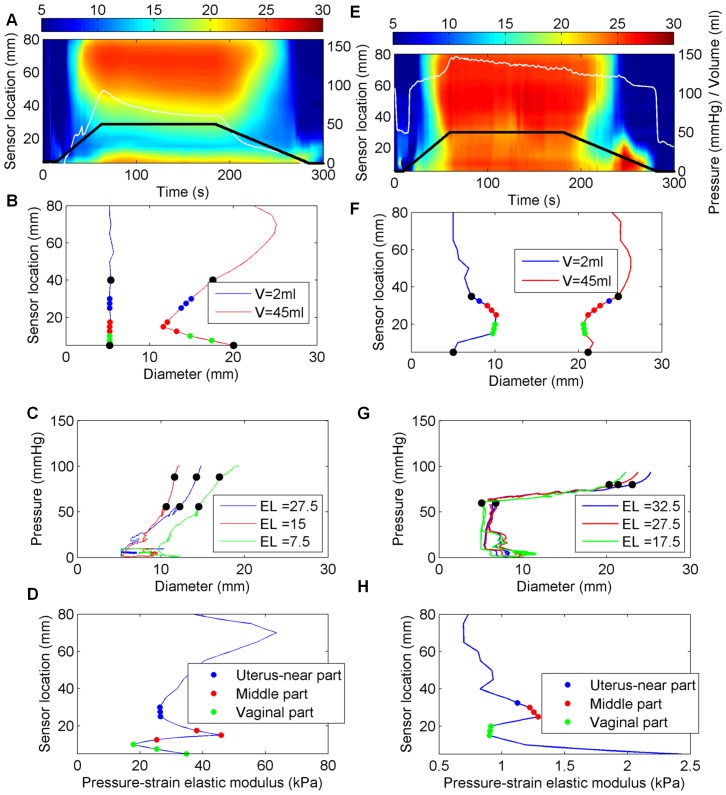
Geometric and biomechanical properties of the uterine cervix of an early-pregnant woman (left column, A–D) and term-pregnant woman with an unripe cervix (right column, E–H). A and E) Spatio-temporal diameter plots. The black line illustrates the volume of the bag (ml), the white line the pressure inside the bag (mmHg), and the colors spanning from blue to red illustrate the magnitude of diameters obtained in the measurement area. B and F) The cervical canal configuration generated at distension volume 2 ml (blue line) and 45 ml (red line). The black dots represent the internal and external cervical os of the uterine cervix. C and G) Pressure-diameter plot. The pressure-diameter relationship for three locations representing the uterus-near part (blue line), the middle part (red line), and the vaginal part (green line) of the uterine cervix. The black dots represent the linear part of the curve. D and H) Pressure-strain elastic modulus plot. The pressure-strain elastic modulus distribution along the cervical canal.

Distension data were obtained from all the included women. The EP_max_ was always found in the middle of the three cervical parts in both early and late pregnancy. Figure 3 shows three-dimensional (3D) reconstructions of the uterine cervix visualized by the EndoFlip at distension volumes 25 ml and 50 ml for an early-pregnant and a term-pregnant woman. The colors from blue to red indicate the increase of the diameter (mm) measured at a specific location along the cervical canal. The narrowest diameter was found in the middle of the canal (blue color, Figure 3). The top of the probe corresponds to sensor numbers 14–16, which were placed in the uterus, and the bottom of the probe corresponds to sensor numbers 1–5, which were placed in the vagina (Figure 3). For the early-pregnant women the diameter of the middle of the cervical canal was unchanged from distension volume 25 to 50 ml, whereas for the term-pregnant women the middle of the canal changed from darker blue color to lighter blue color indicating increasing diameter. Each experiment was also illustrated graphically by spatial-temporal diameter plot (Figure 4–5A and E), volume-diameter plot (Figure 4–5B and F), pressure-diameter plot (Figure 4–5C and G) and pressure-strain elastic modulus plot (Figure 4–5D and H).

**Figure 6:**
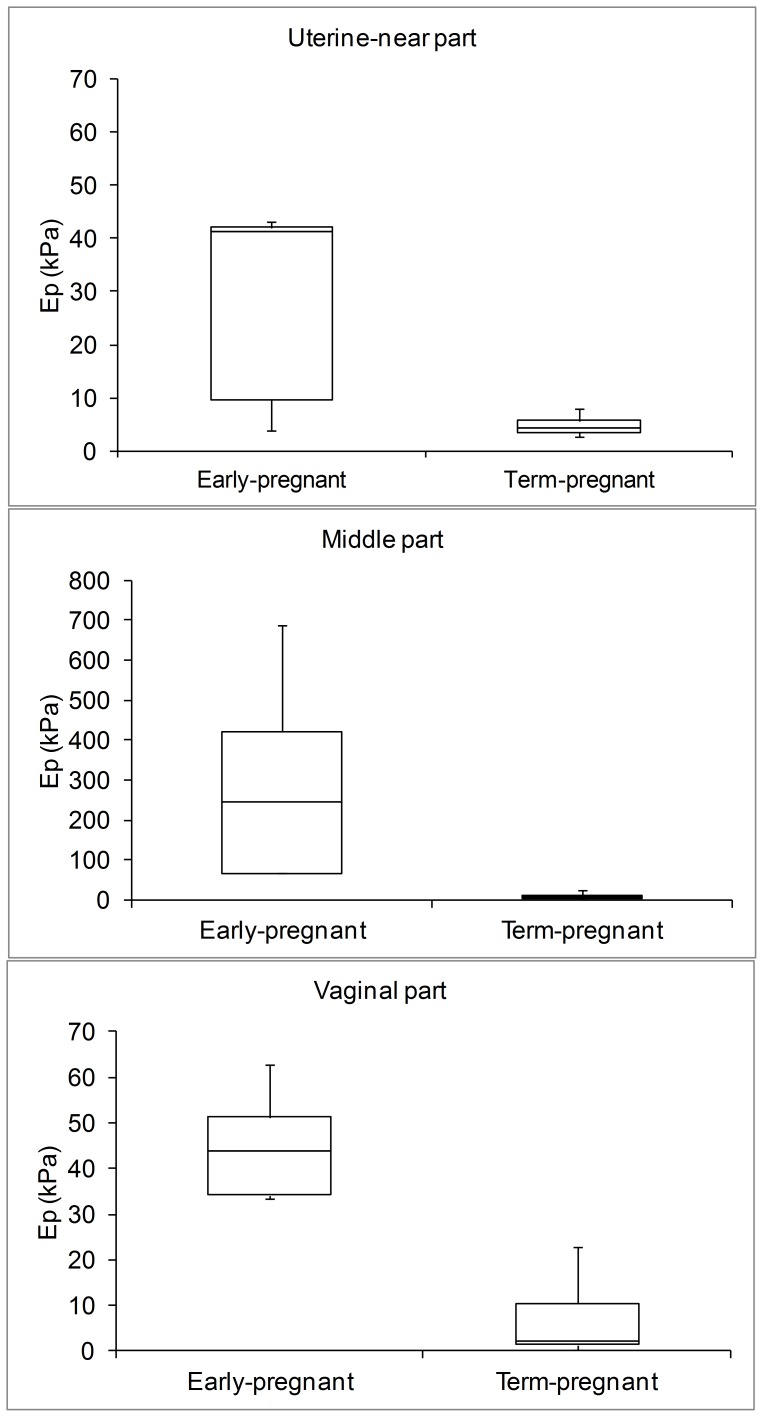
Box-plots showing the pressure-strain elastic modulus of the uterine-near part, the middle part and the vaginal part of the uterine cervix for early and term-pregnant women. The plus whiskers display the upper values within 1.5 times the interquartile range beyond 75th percentile and the minus whiskers, display the minimum value. Difference in EP along the length of the cervix was found for the early-pregnant women.

**Figure 7:**
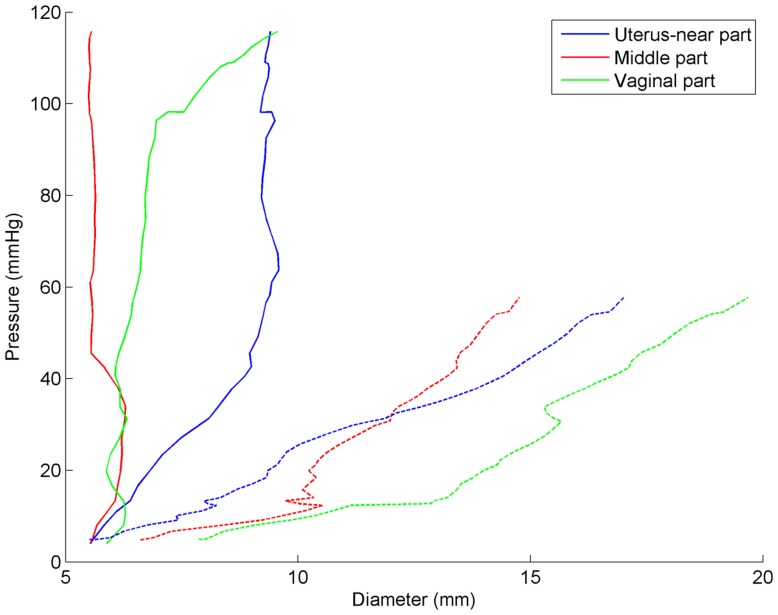
The averaged pressure-diameter curves for early-pregnant (solid lines) and term-pregnant women (dashed lines).

A pronounced difference in the median EP_max_ was found between the early-pregnant women (median 243 kPa, IQR 67–422 kPa) and the term-pregnant women (median 5 kPa, IQR 4–15 kPa) (Figure 6 and Table 1). In the early-pregnant group the middle part of the cervical canal only dilated a few millimetres on average even at a pressure of 120 mm Hg (Figure 7). In contrast, in the term-pregnant women the middle part dilated to an average of 14 mm at a pressure of only 60 mmHg (Figure 7). Among the term-pregnant women the EP parameter from the middle part of the canal differed considerably (EP_max_, range: 0.89–15 kPa, without an outlier of 147 kPa) from unripe (Figure 4, right column) to ripe cervixes (Figure 5). One term-pregnant woman had an unripe cervix, in which only a marginal dilation was observed during inflation, very similar to the uterine cervix of the early-pregnant women (Figure 4, left column). Figure 5 shows results from two term-pregnant women with a medium ripe (left column) and a fully ripe cervix (right column), respectively. The medium ripe cervix dilated from 5 to 12 mm during bag inflation whereas the fully ripe cervix was dilated 10 mm even before bag inflation (Figure 5).

**Table 1:** The pressure-strain elastic modulus (EP) for the uterine-near part, the middle part and the vaginal part of the uterine cervix.

	Early-pregnant women (n = 5)	Term-pregnant women (n = 6)	
Uterine cervix	Median, kPa (IQR)	Median, kPa (IQR)	P-value, unpaired t-test
Uterine-near part	41.3 (9.7–42.1)	4.23 (3.52–6.06)	0.01
Middle part	243 (66.8–422)	5.02 (3.63–15.0)	0.01
Vaginal part	43.7 (34.3–51.3)	2.17 (1.16–13.2)	0.01

Significant difference (one-way ANOVA) was found when comparing the three cervical parts for the early-pregnant women (p = 0.04) whereas axial variation was not found for the term-pregnant women (p = 0.88).

Differences among the three parts of the uterine cervix were found for the early-pregnant women (p = 0.04) (Figure 6 and Table 1) whereas such axial variation was not found in the term-pregnant women (p = 0.88). Positive associations between the median EPs of the uterus-near and middle part (Spearman’s rho = 0.84, p = 0.002), vaginal and middle part (Spearman’s rho = 0.81, p = 0.005), and uterus-near and vaginal part (Spearman’s rho = 0.85, p = 0.001) were found when both groups were analyzed together.

## Discussion

In this study it was possible to obtain geometric profiles of the entire cervix during distension. Differences in the stiffness (EP) of the uterine cervix were found between early (243 kPa) and late (5 kPa) pregnancy. Furthermore, women with a high EP in the vaginal part also had a high EP in the middle part.

A limitation of the inflating procedure of the EndoFlip in the cervix is that the probe could dislocate – sometimes up to 1 cm – during the inflation of the bag. However, this source of error was minimal since we could determine when dislocation happened and correct for it. Computation of mechanical parameters from bag distension data is complex and based on assumptions related to measurement principle, edge effects, equations used, etc. [16]. For this type of mechanical experiment bending (secondary stress) generated from the larger distension of the adjacent vaginal and uterine tissue compared to the cervical canal could influence the measurements at both ends of the cervical canal [17]. Thus, the EP in the vaginal part and the uterine-near part of the uterine cervix may be underestimated. Another limitation of the present study was that Oxytocin was given to the term women and Misoprostol to the early-pregnant women prior to the experiment. These drugs may induce smooth muscle contractions, especially in the uterine-near part of the cervix. Therefore, the mechanical properties that we studied may not be purely passive but may also partly reflect contractile properties.

Despite the limitations of the study, it is to the best of our knowledge the first to evaluate the stiffness of the entire length of the uterine cervix in vivo of pregnant women. It is of interest to notice that the 49 fold decrease in cervical stiffness from early to late pregnancy determined by this study is of similar magnitude as the decrease in stiffness obtained by an in vitro study on biopsies from the vaginal part of the uterine cervix (40 N/mm^2^ in non-pregnant women and 1.84 N/mm^2^ immediately after delivery) [18]. Two studies evaluating the biomechanical properties of the uterine cervix in vivo also found decreasing stiffness (increased distensibility) of the uterine cervix from early to late pregnancy although not as pronounced as in our study [19], [20]. The first study measured the distensibility of the cervical tissue by an aspiration test in vivo. The technique is based on the application of predefined pressure in a pipette (diameter 8 mm) attached to the anterior lip causing tissue deformation [19]. A video camera placed on the pipette films this tissue deformation. The stiffness decreased from 0.04 bar/mm in gestational week 21^+0^ to 0.01 bar/mm in week 36^+0^. The strength of this method is that it is applicable in the clinic and non-invasive. On the other hand, it only evaluated the surface (4 mm) of the uterine cervix. The second study measured the force applied to dilate the uterine cervix with an instrument (“cervicotonometer”) consisting of two branches which were both introduced into the cervical canal [20]. The branches were opened using the thumb and pressing a steel strip at the other end of the instrument. A three-fold increase was found in the “cervical distensibility index” from 22 weeks of gestation (mean 2.8 cm/kg, SD = 0.9 cm/kg) to term (mean 8.1 cm/kg, SD = 3.1 cm/kg). The studies mentioned above have in common that they only evaluated the vaginal part of the uterine cervix, and this might be the reason why they did not find a difference in the stiffness (distensibility) from early to late pregnancy as pronounced as we found.

For the term-pregnant group cervical ripening differed considerably among the women (EP, range: 0.89–15 kPa) excluding an outlier of 147 kPa. This result is similar to that found by the group using the “cervicotonometer” (range: 0.2–12.6 cm/kg) for evaluation of term-pregnant women admitted for routine examination [21]. The spread in the stiffness values found in vitro from post-term cervical biopsies was only between 0.42–3.26 N/mm^2^ [18]. The narrower range in the in vitro study compared with our study may be due to the post-partum women being a more homogenous group compared with our term-pregnant group.

Studies evaluating axial variation in the uterine cervix show diverging results. Histologically, the cervical stroma is dominated by collagen [22], [23] with no differences in the collagen concentration between the two ends [24]. The other large component of the cervical stroma is smooth muscle where the concentration is highest in the uterine-near part with 29% compared to only 6% in the vaginal-part [7]. Another study confirmed the difference although less pronounced (15% in the uterine-near part vs. 8% in the vaginal part) [8]. In vitro testing of the biomechanical properties of biopsies from the uterine-near and vaginal part of non-pregnant cervixes showed no difference in stiffness between the two ends (4.0 N/mm^2^, SD = 0.4 N/mm^2^ vs. 3.5 N/mm^2^, SD = 0.5 N/mm^2^, p = ns) [24]. As the only in vivo method, elastography has assessed the biomechanical properties of the uterine-near and vaginal part of pregnant women [10]. The method is based on manual tissue compression by the ultrasound transducer during ordinary B-mode scan. The authors found increased compressibility of the vaginal part of the cervix compared with the uterine-near part. However, they emphasized that this may be an artifact due to the vaginal part receiving most of the compression by the transducer, and the result might therefore not be trustworthy [10]. This study showed significant difference between the three parts of the uterine cervix with the middle part being stiffer than the two other parts for the early-pregnant women.

Biomechanical testing of the uterine cervix has been evaluated from different clinical perspectives. For instance, two studies have demonstrated that non-pregnant women with a history of preterm birth have increased distensibility of the cervix as compared with control groups. The first study evaluated the pressure-volume relationship assessed by a 4-cm compliant balloon applied within the cervical canal [25] whereas the second study measured the force required to pass a dilator with a diameter of 8 mm through the cervix [26]. A third study conducted by the group evaluating the “cervicotonometer” assessed preterm and post-term delivery in a present pregnancy [27]. They tested women admitted with preterm labor between gestational week 26^+5^ and 35^+0^. The women who delivered before 37^+0^ weeks of gestation had more distensible cervixes (9.99 cm/kg, SD = 2.32 cm/kg) than women who delivered at or after 37^+0^ weeks of gestation (6.92 cm/kg, SD = 2.80 cm/kg). Regarding post-term delivery, they examined pregnant women between gestational weeks 37^+0^ and 40^+5^. Those who ended up having post-term pregnancy (after 41^+0^ weeks of gestation) had less distensible cervixes (4.5 cm/kg, SD = 2.4 cm/kg) than women who delivered at or before 41^+0^ weeks of gestation (6.4 cm/kg, SD = 3.1 cm/kg) (p<0.005). Furthermore, the measurements correlated with the Bishop scores [21].

In conclusion, this new method provides a geometric profile of the cervical lumen during distension and estimates the stiffness along the entire cervical length in vivo with good spatial and temporal resolution. The EndoFlip method may be useful in the evaluation of other methods testing the biomechanical properties of the uterine cervix such as elastografi [10], the collascope [3] and quantitative ultrasound [28]. Another perspective may be the use in non-pregnant women with prior preterm birth. Those with reduced stiffness may benefit from cerclage, whereas those with normal stiffness may benefit from treatment with progesterone.
